# Quality of life of women with breast cancer in a tertiary referral university hospital

**DOI:** 10.1186/s12955-022-01921-1

**Published:** 2022-01-29

**Authors:** Jamilah Yusoff, Aniza Ismail, Mohd Rizal Abd Manaf, Fuad Ismail, Norlia Abdullah, Rohaizak Muhammad, Shahrun Niza Abdullah Suhaimi, Reena Rahayu Mat Zin

**Affiliations:** 1 Ministry of Health Malaysia, Putrajaya, Malaysia; 2 Faculty of Medicine, Universiti Kebangsaan Malaysia, Kuala Lumpur, Malaysia

**Keywords:** Breast cancer, Quality of life, Tertiary hospital

## Abstract

**Background:**

Quality of life (QoL) is one of the treatment outcome measures in patients with breast cancer. In this study, we measured the QoL of women with breast cancer at Universiti Kebangsaan Malaysia Medical Centre (UKMMC) and identified the associated factors.

**Methodology:**

This cross-sectional study was conducted from October 2017 to December 2017 and involved female patients with breast cancer. The QoL scores and domains were determined using the EuroQol EQ-5D-5L, and were presented as the utility value and visual analog scores, respectively.

**Results:**

We recruited a total of 173 women, aged 33–87 years. The median VA score was 80.00 (interquartile range [IQR] 70.00–90.00); the median utility value was 0.78 (interquartile range [IQR] 0.65–1.00. Women who did not take traditional medicine had a higher utility index score of 0.092 (95% CI 0.014–0.171), and women with household income of RM3000–5000 had a higher utility index score of 0.096 (95% CI 0.011–0.180).

**Conclusion:**

Traditional medicine consumption and household income were significantly associated with lower QoL. The pain/discomfort domain was the worst affected QoL domain and was related to traditional medicine use and household income. Addressing pain management in patients with breast cancer and the other factors contributing to lower QoL may improve the QoL of breast cancer survivors in the future.

## Introduction

Breast cancer is the second most common cancer in the world and is the most frequent cancer in women in many parts of the world. An estimated 2.1 million new breast cancer cases were diagnosed in 2018, making it the fifth leading cause of death, with an estimated 627,000 deaths [1]. In Malaysia, breast cancer is on an increasing trend, and remains the cancer with the highest incidence in women. Data from 2020 showed that, in Malaysia, new breast cancer cases numbered the highest (17.3%) compared to other new cancer cases, and numbered the second highest among women (32.9%) [2].

In 2018 a total of 7593 new breast cancer cases in Malaysia compared to colorectal (12%), cervix uteri (7.2%), and other cancers, and breast cancer constitutes the highest mortality rate (age-standardized rate [ASR]: 18.4 per 100,000) [3]. The Malaysian National Cancer Registry reported that the age-standardized (world) incidence of breast cancer in Malaysia in 2018 had increased to 47.5 per 100,000 population compared to that in 2011 (ASR: 31.1 per 100,000) [4].

Breast cancer causes a major psychological impact and stress because it is life-threatening, leads to body image issues resulting from surgical procedures such as mastectomy, the primary treatment is complex (consisting of surgery, chemotherapy, radiotherapy, and endocrine hormonal therapy), hospital visits are frequent, and hospital waiting times are long [5]. Inevitably, hospital visits for chemotherapy, radiotherapy, investigation procedures, and surgery affect patients financially. Social activities such as work, childcare, leisure time, and daily living are disrupted, further adding to the stress and subsequently leading to decreased quality of life (QoL). Furthermore, patients experience adverse effects from the above treatment modalities, subsequently experiencing further increased stress.

Various factors influence QoL in patients with breast cancer. These factors include socioeconomic status, education status, employment status, psychosocial challenges, and the financial factor [6]. Patients with breast cancer may face financial difficulties that can affect their savings and their property. They might struggle to afford basic needs such as food and clothes as a result of the loss of income, health service expenditures, and paid/unpaid work reduction, all of which are the greatest sources of economic burden in patients with breast cancer [7].

The present study was aimed at measuring the QoL of patients with breast cancer who were receiving treatment or on follow-up at Universiti Kebangsaan Malaysia Medical Centre (UKMMC). Specifically, the QoL was measured using the utility value and visual analog (VA) score, and factors affecting QoL, such as sociodemographic, clinical, and financial factors, were examined.

## Methods

### Sample recruitment

This study was conducted at UKMMC, a tertiary teaching public hospital in Kuala Lumpur, Malaysia. The hospital receives referrals from nearby health centers, including those from other states, as it provides specialty services in radiology, pathology, breast surgery, and oncology.

This cross-sectional study was conducted in 2017. Before the study commenced, ethics approval was obtained from the UKM ethical committee. Written informed consent was obtained from all participants. Participants were recruited by universal sampling among women aged ≥ 18 years who had been diagnosed with breast cancer and who had visited UKMMC facilities from October 2017 to December 2017 as outpatients or inpatients.

Outpatients were identified when they registered at the oncology or surgical clinic. Day care admission consisted of patients who came for chemotherapy, while inpatients were patients with breast cancer who were admitted to the surgical wards.

We excluded men with breast cancer, or foreigners and women who unwilling or unable to consent and unable to complete the questionnaire.

### Data collection

The QoL was assessed with a self-administered questionnaire using the EQ-5D-5L, which is available in three languages (Malay, English, Chinese). The questionnaire has been validated by the Euro QoL Group, and consists of the EQ-5D-5L descriptive system and the visual analogue score (VAS) [8]. The EQ-5D-5L descriptive system consists of five domains (mobility, self-care, regular activity, pain/discomfort, anxiety/depression), with five levels of perceived health problems from “no problem” (level 1) to “extreme problem” (level 5).

The VA scale is a simple tool for describing the general health status, and is scaled from 0 (worst health status) to 100 (best health status). The EQ-5D-5L descriptive system scores (multiple values for five domains) were converted into one single health state score for each domain, termed the utility value. The value of 0 represented a “dead” state, and 1 a “full health” state. The dependent variable was the VAS and utility value. The VAS and utility values were according to that in two local studies [9, 10].

### Data analysis

Data entry was performed using Excel sheets, and data analysis was carried out with SPSS 23. Descriptive analysis (Table 1) included the participants’ sociodemographic characteristics such as age, marital status, ethnicity, education level, employment status, presence of concurrent illness, duration of illness, frequency of clinic visits, treatment received, individual and family monthly incomes.

**Table 1:** Characteristics of the respondents

Characteristic	Number (n = 173)	Percentage (%)
1. Sociodemographic		
Age (years)		
30–39	7	4.0
40–49	34	19.7
50–59	61	35.3
60–69	57	32.9
> 70	14	8.1
Marital status		
Single	14	8.1
Married	139	80.3
Divorced	9	5.2
Widowed	11	6.4
Ethnicity		
Malay	117	67.6
Chinese	44	25.4
Indian	12	6.9
Others	0	0.0
Level of formal education		
None	8	4.6
Primary	32	18.5
Secondary	77	44.5
Tertiary	56	32.4
Employment status		
Never employed	37	21.4
Unemployed	32	18.5
Self employed	4	2.3
Part time	3	1.7
Full time	48	27.8
Pensioner	49	28.3
Presence of concurrent illnesses		
Yes	99	57.2
No	74	42.8
2. Duration and Treatment		
Duration of breast cancer		
< 1 year	40	23.1
≥ 1- 5 years	82	47.4
≥ 5- 10 years	26	15.0
≥ 10–15 years	16	9.2
≥ 15 years	9	5.2
Treatment received		
Endocrine (Hormonal) therapy		
Yes	121	69.9
No	52	30.1
Chemotherapy		
Yes	137	79.2
No	36	20.8
Radiotherapy		
Yes	121	69.9
No	52	30.1
Surgery		
Yes	163	94.2
No	10	5.8
Traditional medicine		
Yes	41	23.7
No	132	76.3
3. Financial monthly income		
Individual (RM)		
≤ 500	64	37.0
> 500–1000	24	13.9
> 1000–5000	68	39.3
> 5000	17	9.8
Household (RM)		
≤ 1000	32	18.5
> 1000–3000	52	30.1
> 3000–5000	30	17.3
> 5000–10,000	47	27.2
> 10,000	12	6.9
Savings affected		
Yes	42	24.3
No	131	73.4
Problem buying basic needs		
Yes	19	11.0
No	154	89.0
Property affected		
Yes	12	6.9
No	161	93.1

The duration of illness was calculated in ears from the time of diagnosis until December 2017. Treatment received referred to the therapy received since diagnosis, i.e., endocrine (hormonal) therapy, chemotherapy, radiotherapy, surgery, or traditional medicine.

The association of the QoL utility value and VAS with the sociodemographic factors, clinical characteristics, and financial factors was examined using the Mann–Whitney U test and Kruskal–Wallis test. Higher utility and VAS values indicated better QoL. The domain affecting the independent variables was examined with similar tests; higher values indicated worse conditions. Statistical significance was set at *p* ≤ 0.05. Multivariate analysis via multiple linear regression (MLR) was used for the categorical independent variables and all dummy variables created were included in the MLR model.

## Results

A total of 189 women with breast cancer were identified, and 173 were successfully recruited (response rate: 93.0%). Ten women declined to participate, while three provided incomplete information. Another three women were excluded for other concurrent cancers, i.e., ovarian and thyroid cancer. The respondents’ characteristics are summarized in Table 1.

More than half of the patients were in the 50–69-year age range (68.2%) and 19.7% were in the 40–49-year age range. The youngest patient was 33 years old, while the oldest was 87 years old. Most of the patients were married (80.3%), while the rest were single, divorced, or widowed. A small number of patients (4.6%) did not have formal education, while 18.5% had received primary education, 44.5% had secondary education, and 32.4% had tertiary education. More than half of the patients (57.2%) had a concurrent illness other than breast cancer.

Up to 23.1% of the patients had been diagnosed less than a year ago, 47.4% of the patients had been diagnosed 1–5 years ago, 15.0% had been diagnosed 5–10 years ago, and 5.2% had been diagnosed > 15 years ago. Most of the patients received surgical treatment (94.2%), followed by chemotherapy (79.2%), endocrine therapy (69.9%), and radiotherapy (69.9%); 23.7% used traditional or complementary medicine such as herbs, homeopathy, and acupuncture.

A significant number of the patients (37.9%) had a monthly income of RM1000–5000, 13.9% earned > RM5000, 37% earned < RM500, and 17.3% had no income. The median monthly income was RM1000 (RM0–10,000), while the median household income was RM3200 (RM0–39,000). About 18.5% of the patients were very poor, with a household income of < RM1000, and 30.1% were poor, with a household income of < RM3000. This indicated that almost 50.0% of the patients were socioeconomically poor. The middle-income category, consisting of about 17.3% of the patients, had a monthly household income of RM3000–5000, while 27.2% of the patients had a monthly household income of RM5000–10,000. A small group of patients (6.9%) comprised the high-income group, earning > RM10,000 monthly; the highest monthly household income was RM39,000. About 11.0% of the patients had problems buying necessities; 24.3% reported that their savings were affected, and 6.9% claimed that their property was affected due to breast cancer.

## Quality of life

The EQ-5D-5L showed that, except the pain/discomfort domain, most of the patients had no difficulty in the other QoL domains (Table 2). More than 70% of the patients perceived no problems regarding mobility, self-care, normal activities, and anxiety/depression, while only 51.4% perceived no pain/discomfort. About 35.8% of the patients reported minimal pain/discomfort, 9.2% had moderate pain/discomfort, and 3.5% had severe pain/discomfort. Less than 3% of the patients faced extreme difficulty in mobility (1.2%), self-care (0.6%), normal activities (2.9%), and extreme anxiety/discomfort (0.6%). However, no patient experienced extreme pain/discomfort. The mean and median VAS was 79.65 (standard deviation [SD] 15.985) and 80.00 (interquartile range [IQR] 70.00–90.00), respectively, with a minimum score of 10 and a maximum score of 100. The mean and median utility value was 0.78 (SD 0.220) and 0.78 (interquartile range [IQR] 0.65–1.00), respectively.

**Table 2:** Quality of life of respondents

EQ-5D-5L descriptive system	Frequency	Percentage (%)
Domain		
Mobility		
1	126	72.8
2	27	15.6
3	13	7.5
4	5	2.9
5	2	1.2
Self-care		
1	161	93.1
2	7	4.0
3	3	1.7
4	1	0.6
5	1	0.6
Normal activities		
1	133	76.9
2	18	10.4
3	12	6.9
4	5	2.9
5	5	2.9
Pain/discomfort		
1	89	51.4
2	62	35.8
3	16	9.2
4	6	3.5
5	0	0.0
Anxiety/depression		
1	126	72.8
2	40	23.1
3	6	3.5
4	0	0.0
5	1	0.6

EQ-5D-5L component	Median (IQR)	Mean (SD)
VAS	80.00 (70.00–90.00)	79.65 (15.985)
Utility value	0.78 (0.65–1.00)	0.78 (0.220)

Table 3 summarizes the analysis of the association between the VAS and utility value with the independent variables. Ethnicity and the presence of concurrent illnesses were significantly associated with the VA score. Malay and Indian patients had significantly higher VAS compared to Chinese patients (χ^2^ = 9.079, *p* = 0.011). Patients with concurrent illnesses had significantly lower VA scores (χ^2^ = − 2.132, *p* = 0.033). Traditional medicine use and family income was significantly associated with the utility value. Patients who took traditional medicine had significantly lower utility values (z = − 2.480, *p* = 0.013), while patients with household income between > RM3000 and RM5000 had significantly higher utility values compared to the other income groups (χ^2^ = 10.230, *p* = 0.037).

**Table 3:** Association of utility score and VA score

Characteristic	Number (n = 173)	Utility value			VAS		
		Mean rank (sum of ranks)	z/χ^2^	*p*-value	Mean rank (sum of ranks)	z/χ^2^	*p*-value
Sociodemographic							
Age (years old)*							
30–39	7	72.93	2.304	0.680	73.93	1.505	0.826
40–49	34	91.24			92.32		
50–59	61	91.92			89.35		
60–69	57	81.11			82.51		
> 70	14	86.32			82.64		
Marital status*							
Single	14	85.61	0.400	0.940	70.54	3.302	0.347
Married	139	87.18			88.39		
Divorced	9	79.28			73.33		
Widowed	11	92.86			91.59		
Ethnicity*							
Malay	117	87.47	0.884	0.643	94.20	9.079	0.011^‡^
Chinese	44	82.89			67.83		
Indian	12	97.50			87.13		
Others	0	0			0		
Formal education*							
None	8	63.69	3.555	0.314	88.00	0.960	0.81
Primary	32	79.34			79.48		
Secondary	77	88.16			89.60		
Tertiary	56	93.12			87.57		
Employment status*							
Never employed	37	87.77	4.345	0.501	94.89	1.671	0.893
Unemployed	32	82.48			82.83		
Self employed	4	66.75			81.38		
Part time	3	127.33			83.33		
Full time	48	93.48			88.79		
Pensioner	49	82.29			82.69		
Presence of concurrent illness^†^							
Yes	99	83.41	− 1.121	0.262	80.07	− 2.132	0.033^‡^
No	74	91.80			96.28		
Duration and treatment							
Duration of BC*							
< 1 year	40	82.96	1.163	0.884	89.15	4.299	0.367
≥ 1–5 years	82	88.18			90.80		
≥ 5–10 years	26	90.10			70.73		
≥ 10–15 years	16	80.13			80.91		
≥ 15 years	9	97.44			100.67		
Treatment received							
Endocrine (Hormonal) therapy^†^							
Yes	121	89.24	− 0.922	0.357	87.02	− 0.008	0.993
No	52	81.80			86.95		
Chemotherapy^†^							
Yes	137	88.36	− 0.720	0.472	89.17	− 1.124	0.261
No	36	81.81			78.75		
Radiotherapy^†^							
Yes	121	89.43	− 1.000	0.317	89.78	− 1.126	0.260
No	52	81.36			80.54		
Surgery^†^							
Yes	163	88.02	− 1.108	0.268	86.44	− 0.596	0.551
No	10	70.45			96.05		
Traditional medicine^†^							
Yes	41	70.54	− 2.480	0.013^‡^	87.80	− 0.119	0.905
No	132	92.11			86.75		
Financial monthly income							
Individual* (RM)							
≤ 500	64	87.13	0.733	0.866	84.46	2.159	0.540
> 500–1000	24	81.33			76.44		
> 1000–5000	68	87.00			92.58		
> 5000	17	94.53			89.15		
Household* (RM)							
≤ 1000	32	71.63	10.230	0.037^‡^	79.73	2.692	0.611
> 1000–3000	52	91.38			86.07		
> 3000–5000	30	106.48			86.17		
> 5000–10,000	47	84.88			95.94		
> 10,000	12	68.58			77.50		
Savings affected^†^							
Yes	42	85.74	− 0.193	0.847	82.60	− 0.663	0.507
No	131	87.40			88.41		
Problem buying basic needs^†^							
Yes	19	83.53	− 0.330	0.742	73.45	− 1.265	0.206
No	154	87.43			88.67		

*Variables were tested by Kruskal Wallis test and presented as Chi-square

^†^Variables were tested by Mann Whitney U test and presented as z value

^‡^Significant *p*-value at *p* < 0.05 (2 tailed)

Table 4 summarizes the association between the EQ-5D-5L domains and other variables. The mobility and anxiety/depression domains were not significantly associated with any variable. However, the self-care domain was significantly associated with hormonal therapy (z = − 2.165, *p* = 0.030) and problems buying basic needs (z = − 2.591, *p* = 0.010). The usual activity domain was significantly associated with household income (χ^2^ = 9.967, *p* = 0.041). The pain/discomfort domain was significantly associated with traditional medicine use (z = − 3.108, *p* = 0.002) and household income (χ^2^ = 12.845, *p* = 0.012).

**Table 4:** EQ-5L-5D domains and association with socio-demography, duration & treatment, and financial monthly income

Characteristic	Mobility		Self-care		Usual activity		Pain/discomfort		Anxiety / depression	
	Mean rank	z/ χ^2^ (*p*-value)	Mean rank	z/ χ^2^ (*p*-value)	Mean rank	z/ χ^2^ (*p*-value)	Mean rank	z/ χ^2^ (*p*-value)	Mean rank	z/ χ^2^ (*p*-value)
Sociodemographic										
Age (years old)^1^										
30–39	78.57	3.546 (0.471)	93.00	1.282 (0.864)	102.71	3.778 (0.437)	110.07	2.584 (0.630)	114.71	3.777 (0.437)
40–49	80.69		86.09		82.88		88.74		86.82	
50–59	84.16		85.21		82.84		85.17		85.65	
60–69	92.34		88.60		92.51		87.41		85.10	
> 70	97.18		87.50		84.86		77.54		87.21	
Marital status^1^										
Single	89.11	1.643 (0.650)	87.57	6.601 (0.086)	93.36	1.663 (0.645)	89.11	5.124 (0.163)	77.00	1.729 (0.631)
Married	87.27		85.27		85.39		88.21		88.40	
Divorced	72.00		100.78		88.61		99.67		91.17	
Widowed	93.14		96.91		97.95		58.73		78.59	
Ethnicity^1^										
Malay	86.59	0.052 (0.974)	87.62	3.237 (0.198)	85.48	0.618 (0.734)	85.97	0.221 (0.895)	89.12	2.571 (0.277)
Chinese	88.16		83.07		90.36		88.57		85.90	
Indian	86.71		95.42		89.50		91.29		70.42	
Others	0		0		0		0		0	
Formal education^1^										
None	109.25	4.301 (0.231)	102.88	4.439 (0.218)	100.13	4.570 (0.206)	84.44	0.468 (0.926)	94.63	0.569 (0.904)
Primary	93.41		86.50		97.34		85.88		83.81	
Secondary	85.16		86.65		83.53		89.57		87.86	
Tertiary	82.69		85.50		83.98		84.47		86.55	
Employment status^1^										
Never employed	84.76	4.083 (0.538)	81.00	8.187 (0.146)	88.85	1.676 (0.892)	84.27	7.017 (0.219)	84.31	4.182 (0.524)
Unemployed	92.11		89.19		92.63		87.97		95.02	
Self employed	89.88		103.25		93.00		133.00		63.50	
Part time	63.50		81.00		92.17		45.00		88.19	
Full time	80.69		84.50		83.18		85.29		85.99	
Pensioner	92.74		91.59		84.87		88.92			
Presence of concurrent illness^2^										
Yes	91.01	− 1.560 (0.119)	87.99	− 0.683 (0.495)	90.16	− 1.300 (0.194)	87.82	− 0.277 (0.780)	90.08	− 1.207 (0.227)
No	81.64		85.68		82.78		85.90		82.88	
Duration and treatment										
Duration of BC^1^										
< 1 year	85.35	0.441 (0.979)	85.33	7.576 (0.108)	88.68	6.207 (0.184)	94.88	2.949 (0.566)	96.35	6.482 (0.166)
≥ 1- 5 years	87.70		89.44		92.28		81.61		83.62	
≥ 5- 10 years	89.35		81.00		73.71		88.52		79.46	
≥ 10–15 years	87.41		81.00		77.38		94.78		99.81	
≥ 15 years	80.50		100.22		86.94		82.89		75.28	
Treatment received										
Hormonal therapy^2^										
Yes	85.90	− 0.717 (0.474)	84.62	2.165 (0.030)*	85.23	− 0.963 (0.336)	87.64	− 0.282 (0.778)	85.03	− 1.018 (0.309)
No	90.25		92.54		91.13		85.52		91.59	
Chemotherapy^2^										
Yes	85.18	− 1.198 (0.231)	87.30	− 0.348 (0.728)	86.34	− 0.456 (0.648)	87.83	− 0.472 (0.637)	88.16	− 0.767 (0.443)
No	93.94		85.86		89.50		83.83		82.58	
Radiotherapy^2^										
Yes	85.33	− 0.855 (0.393)	85.31	− 1.534 (0.125)	84.13	− 1.557 (0.119)	86.99	− 0.004 (0.997)	86.05	− 0.491 (0.623)
No	90.88		90.92		93.67		87.02		89.21	
Surgery^2^										
Yes	86.32	− 0.925 (0.355)	86.29	− 1.699 (0.089)	86.60	− 0.573 (0.567)	87.02	− 0.025 (0.980)	86.77	− 0.310 (0.756)
No	98.10		98.50		93.50		86.65		90.70	
Traditional medicine^2^										
Yes	95.34	− 1.564 (0.118)	87.39	− 0.130 (0.897)	93.30	− 1.251 (0.211)	106.20	− 3.108 (0.002)*	93.05	− 1.142 (0.254)
No	84.41		86.88		85.04		81.04		85.12	
Financial factor										
Individual monthly income^1^ (RM)										
≤ 500	89.64	1.559 (0.669)	87.83	1.888 (0.596)	89.98	1.453 (0.693)	81.06	4.493 (0.213)	84.45	0.986 (0.805)
> 500–1000	82.46		84.83		85.17		103.35		92.13	
> 1000–5000	88.32		88.49		87.05		85.60		88.59	
> 5000	78.18		81.00		78.15		91.85		83.03	
Household income (RM)^1^										
≤ 1000	89.93	1.357 (0.852)	94.91	7.515 (0.111)	100.13	9.967 (0.041)*	96.61	12.845 (0.012)*	91.59	3.895 (0.420)
> 1000–3000	84.37		85.85		89.96		83.81		83.16	
> 3000–5000	82.02		81.00		72.03		66.43		82.87	
> 5000–10,000	89.91		88.26		83.20		89.83		86.16	
> 10,000	92.33		81.00		91.46		115.54		105.00	
Savings affected^2^										
Yes	89.30	− 0.438 (0.662)	85.24	− 0.595 (0.552)	86.17	− 0.168 (0.867)	90.64	− 0.599 (0.549)	90.29	− 0.630 (0.529)
No	86.26		87.56		87.27		85.83		85.95	
Problem buying basic needs^2^										
Yes	87.90	− 0.109 (0.913)	99.37	− 2.591 (0.010)*	95.95	− 1.119 (0.263)	96.16	− 0.935 (0.350)	82.37	− 0.551 (0.582)
No	86.89		85.47		85.90		85.87		87.57	

*Significant *p*-value at *p* < 0.05 (2 tailed)

^1^ Variables were tested by Kruskal Wallis test and presented as Chi-square

^2^ Variables were tested by Mann Whitney U test and presented as z value

All significant variables in univariate analysis were further analyzed by multivariate analysis using MLR. The MLR for categorical independent variables was performed by creating dummy variables, which were then tested in the model. The final model revealed traditional medicine and family income RM3000–5000 were significantly associated with the utility index. Women who did not take traditional medicine had a higher utility index score of 0.092 (95% CI 0.014–0.171) compared to those taking traditional medicine. Women with household income between RM3000–5000 had a higher utility index score of 0.096 (95% CI 0.011–0.180) compared to women with household income more than RM 10000 (Table 5).

**Table 5:** Significant determinants of the utility index among women with breast cancer

Variables	MLR					
	Unstandardized coefficients		Standardized coefficients beta	95% CI	t-stat	*p* value
	B^b^	Std error				
(Constant)	0.691	0.035	0.052	(0.621–0.761)	19.534	0.000
Traditional medicine						
No	0.092	0.04	0.179	(0.014–0.171)	2.324	*0.021
[Yes]	–	–	–	–	–	–
Household income (RM)						
3000–5000	0.096	0.043	0.173	(0.011–0.180)	2.243	*0.026
[> 10000]	–	–	–	–	–	–

Dependent variable = utility index; [] = Reference; ^b^ = crude regression coefficient

*p < 0.05; *MLR* Multiple Linear Regression (forward MLR was applied); R sq = 0.064; R-sq (adj) = 0.052

## Discussion

In this study, the median VA and utility score was 80.00 and 0.78, respectively. The mean VA score (79.65, SD 5.985) was low compared to Malaysian population score (85.52, SD 12.3) [10]. A previous study that used the same EuroQol questionnaire reported a median utility score of 0.691 [11], which was lower because the authors had sampled patients with advanced breast cancer. However, the QoL in the present research was higher, as the sample consisted of patients with breast cancer of all stages.

An earlier study carried out in 2014 at UKMMC using the European Organization for Research and Treatment of Cancer (EORTC) QLQ-C30 and the breast cancer-specific supplementary module QLQ-BR23 showed a global health status/mean QoL score of 67.81 (SD 18.92), with the greatest impact on emotional functioning [12]. The study showed that UKMMC patients with breast cancer experienced tension, depression, and irritability. The difference in the QoL levels of these two studies could be attributed to the multiple QoL measurement tools and the different methods used. In fact, the absence of a control group could be another limitation.

In the present study, the patients aged 30–39 years had worse QoL due to pain/discomfort as compared to the patients in other age groups, which significantly impaired their daily activities and affected their anxiety/depression domain. These patients had more reduction in mobility with the increase in age. Conversely, several studies have reported that younger women with breast cancer had restrictions in various QoL compared to older women [13–15]. Even though the domains affected younger and older patients with breast cancer differently, there was no significant association with QoL, and no domain was significantly related to age.

A previous study in Malaysia showed that Chinese female patients with breast cancer have a better QoL than patients of other races [16]. The authors used the EORTC QLQ-C30 and QLQ-BR23 modules, which revealed that Chinese patients had better QoL in both the functional and symptom scales. Our results are contradictory to that previous finding, showing that the VA score was significantly higher among Malay (94.20) and Indian patients (87.13) as compared to Chinese patients (67.83). Here, we analyzed the EQ-5D-5L domains to determine their presence between races, and found a significant association. The different QoL between the races may be explained by the difference in social and cultural behavior, socioeconomic status, and psychological and environmental factors. In the US, QoL measures among breast cancer survivors show that Black women demonstrated significantly higher levels of distress involving financial problems as compared to their white or Hispanic counterparts [17]. In Lebanon, Iraqis had a lower QoL score compared to Lebanese and other races [18]. However, the QoL in the present study was measured by the EQ-5D-5L, which is a general tool for measuring QoL. It does not refer to symptoms related to breast cancer, and it is also difficult to compare the QoL domains specifically used for breast cancer, such as the EORTC QLQ-C30 and its supplementary QLQ-BR23. Thus, the different findings obtained in this study need to be interpreted with care.

In the present study, marital status was not significantly associated with QoL. This finding is in accordance a report that being married, single, or widowed had equal impact on QoL [19]. Single women or widows/divorcees can obtain help and support from their close family members, which improves their emotional and physical wellbeing, and subsequently their QoL. However, marital status has been significantly associated with higher QoL [20]. The results of that study also showed that patients without concurrent illnesses had better QoL than patients with concurrent illnesses, which was supported by the study of Claessens et al. [21]. Patients with concurrent illnesses in addition to breast cancer are probably more distressed, which may lower their QoL. In contrast to this finding, another study reported no significant association between QoL and the presence of concurrent illnesses [22].

Here, the use of traditional medicine/treatment was significantly associated with the utility score. Patients who received traditional treatment had significantly lower QoL. We found that women who did not take traditional medicine had a higher utility index score of 0.092 (95% CI 0.014–0.171). However, we did not include how and why the traditional medicine affected their QoL in this study. Our study did not demonstrate any association of QoL score with the duration of cancer diagnosis or type of treatment received. In contrast, only one study from China reported that traditional Chinese medicine treatment for breast cancer was associated with better scores of QoL measures compared to that for chemotherapy [23]. Several studies have also demonstrated similar findings of no significant association between QoL and treatment type [19, 22, 24]. No significant difference has been found between complementary and alternative medicine (CAM) users and non-CAM users [24]. Data on the type of surgery also did not show any difference in QoL; patients who underwent mastectomy and breast-conserving surgery had similar QoL [25].

Almost half of the patients in the present study were of low socioeconomic status, with a monthly household income below RM1000. We postulated that the QoL would be lowest among these patients. This was based on evidence from several studies showing that financial difficulties significantly lowered the QoL of patients with breast cancer [18, 22]. Statistically, we found that QoL was significantly lower among the low-income group. However, low QoL was also present in the very high-household income group, i.e., > RM10,000. Patients with a household income between > RM3000 and RM5000 had a significantly higher utility index score of 0.096 (95% CI 0.011–0.180). In contrast, with higher household income, the presence of medical insurance plans with low co-payment has been associated with better QoL measures [24]. Among the EQ-5D-5L domains, usual activity was the most affected in the very low-socioeconomic status group (household income < RM1000), while pain/discomfort was most affected in the very high-socioeconomic status group (household income > RM10,000).

The EQ-5D-5L showed that, except the pain/discomfort domain, the majority of patients had no difficulty in all other domains. The pain/discomfort domain was significantly associated with traditional medicine use and household income. Other than insomnia and fatigue, pain was the most common symptom reported in QoL studies [26]. Pain and insomnia are the most affected QoL domains in patients with breast cancer, and are not restricted to patients who had received adjuvant chemotherapy and adjuvant radiotherapy [27].

The pain/discomfort domain not only predominantly affected young breast cancer survivors, but was also the worst QoL outcome for all age groups. Moreover, pain/discomfort and anxiety/depression were the most affected QoL domains among breast cancer survivors as compared to their age-matched general population [13]. CAM users experience higher systemic therapy adverse effects such as pain and discomfort. This is probably why these patients seek CAM. CAM users have a significantly higher financial burden as compared to non-CAM users [28].

A systematic review of complementary and alternative treatments has shown that the most common type of CAM used by patients with breast cancer are herbs, vitamins, and food supplements. Younger and educated women are more likely to use CAM [29]. The reasons stated for using CAM were preventing cancer recurrence, curing cancer, and treating conventional adverse effects. However, the use of unprescribed medication such as herbs may lead to drug interactions and unproven efficacy, which may have detrimental effects. On the other hand, the advantages of CAM are that it is cost-effective or cost-saving because it avoids high technology, is non-invasive, encourages healthy lifestyle changes, and is reduces cost in several avenues, i.e., it replaces usual conventional therapy and lowers future healthcare utilization [30].

The limitation of this study is that most of the participants were outpatients and almost 80.0% of them had been diagnosed with breast cancer more than a year prior. In patients with breast cancer, mental functioning is worse during hospitalization than when in surgery; however, it improves over time [31, 32]. The initial 1 year of treatment that consists of surgery, chemotherapy, and radiotherapy is considered the most challenging period. Thus, most patients would have already experienced the difficult times, and this includes the main treatment. In the present study, we did not use the EORTC QLQ-C30 and QLQ-BR23 tools. Rather, we selected the EQ-5D-5L because the objective of the study was to determine the general QoL of a specific population, and not the disease- or treatment-related QoL of the patients.

## Conclusion

Patients with breast cancer treated at UKMMC generally had higher QoL compared to the patients in a 2014 study [12]. The pain/discomfort domain was the worst QoL domain. Traditional medicine use and household income influenced the QoL outcome. Strengthening pain management in patients with breast cancer and greater consideration of the factors contributing to lower QoL may improve the QoL of breast cancer survivors in the future. It is recommended that future studies explore the impact of traditional medicine on the QoL among patients with breast cancer.

## Data Availability

Not applicable.
